# Virgin Camellia Seed Oil Improves Glycolipid Metabolism in the Kidney of High Fat-Fed Rats through AMPK-SREBP Pathway

**DOI:** 10.3390/nu15234888

**Published:** 2023-11-23

**Authors:** Qinhe Zhu, Guihui Li, Li Ma, Bolin Chen, Dawei Zhang, Jing Gao, Senwen Deng, Yongzhong Chen

**Affiliations:** 1 National Engineering Research Center of Oiltea Camellia, State Key Laboratory of Utilization of Woody Oil Resource, Hunan Academy of Forestry, Shao Shan South Road, No. 658, Changsha 410004, China; zhuqinhe@mail.hnust.edu.cn (Q.Z.); liguihui@mail.hnust.edu.cn (G.L.); supermarry1@hnlky.cn (L.M.); zhangdawei.hnust@foxmail.com (D.Z.);; 2 Hunan Key Laboratory of Economic Crops Genetic Improvement and Integrated Utilization, School of Life and Health Sciences, Hunan University of Science and Technology, Xiangtan 411201, China; 3 Co-Innovation Center for the Sustainable Forestry in Southern China, Nanjing Forestry University, Nanjing 210037, China

**Keywords:** camellia seed oil, oil refining, glycolipid metabolism, kidney, energy homeostasis

## Abstract

Camellia seed oil (CO) is used as edible oil in southern China because of its excellent fatty acid composition and abundant bioactive compounds. Chronic kidney disease (CKD) is one of the most common chronic degenerative diseases in China, and active compounds in vegetable oil, like virgin olive oil, have been demonstrated to be efficacious in the management of CKD. In this study, virgin CO was refined using a standard process. The refining had minimal impact on the fatty acid composition, but significantly reduced the presence of bioactive compounds like polyphenols in CO. Sprague-Dawley (SD) rats fed with high fat diet (Group G) were treated with either virgin (Group Z) or refined CO (Group R). The oral administration of CO alleviated lipid accumulation and decreased body and kidney weight gain. Furthermore, treatment with virgin CO increased the renal ATP content. The renal expression levels of AMPK and key enzymes involved in fatty acid oxidation (CPT-1 and ACOX1) and glycolysis (HK, PFK, PK and GAPDH) were up-regulated in Group Z, thereby enhancing the ATP production. Virgin CO treatment downregulated the expression level of SREBP2 and its downstream target genes, such as ACC, FAS, and HMGCR, which reduced lipid synthesis. These findings indicate that virgin CO improves glycolipid metabolism and restores energy homeostasis in the kidneys of rats fed with a high-fat diet by modulating the AMPK–SREBP-signaling pathway, suggesting the potential of active compounds in virgin CO for managing the renal failure associated with glycolipid dysmetabolism.

## 1. Introduction

*Camellia oleifera* Abel, also known as oilseed camellia, is a subtropical evergreen shrub, or small arbor, and belongs to the genus *Camellia* in the family Theaceae [1]. Oilseed camellia, oil palm, olive, and coconut (*Cocos nucifera*) are known as the world’s four major woody oil crops [2,3]. In 2020, the planting area of oilseed camellia in China was 4.46 million hectares, and the production of camellia seed oil (CO) was 720,000 tons (http://www.forestry.gov.cn/main/586/20211103/084728001949510.html, accessed on 28 September 2023). The fatty acid profile of CO is similar to that of olive oil, as both have a high proportion of unsaturated fatty acids (up to 90%) [4]. CO contains abundant bioactive compounds, such as squalene, phytosterols, polyphenols, and tocopherol [5]. It has been highly recommended by the Food and Agriculture Organization of the United Nations for edible use. Additionally, it has been widely used in cosmetic and medicinal fields in China [1,6,7].

CO is commonly extracted from the seeds of *C. oleifera* and its related species using methods like mechanical pressing extraction (MPE) or solvent extraction (SE) [5,8]. However, the crude oil was observed to have inferior quality due to various factors such as the residues of hazardous substances [9,10]. To meet the national standards, the crude oil must be refined, especially those oils obtained by hot pressing extraction (HPE) and SE [11,12]. It was generally considered that the refining processes have limited effects on the fatty acid composition of the oil products [13,14]. However, the bioactive compounds in CO are reduced during the refining process because of the frequently-observed phenomenon of over-refining. For example, 67.24% of the vitamin E and 69.61% of the squalene in CO obtained by HPE can be lost during refining [13]. In another study, the loss of 37.30% of vitamin E, 99.54% of the total polyphenols, 45.60% of squalene, and more than 30% of the steroids were lost [14]. Moreover, over-refining under extreme conditions such as high temperatures can also induce the formation of toxic and harmful compounds such as *trans*-fatty acids (TFA), 3-monochloropropane-1,2-diol (MCPD), and glycidyl fatty acid esters, which can trigger various health problems including heart disease, diabetes, and inflammation [15,16]. Also, the refining processes can cause the loss of certain amount of oil and consume a large amount of raw materials such as clay [15].

Moderate refining technology has been put forward in the academic and industrial fields to reduce the loss of active substances [17]. However, the research on the exact role of active compounds in CO is limited. CO mainly contains unsaturated fatty acids such as oleic acid and linoleic acid. These acids can effectively reduce serum total cholesterol, triglycerides, and low-density lipoprotein cholesterol, while increasing high-density lipoprotein cholesterol, and help to reduce lipid accumulation in the liver [18,19]. Various active ingredients such as polyphenols in CO can influence lipid metabolism, adipocyte differentiation, intestinal absorption, and gut microbes, thereby regulating lipid metabolism in the body [20,21]. They may interact with fatty acid in CO and influence various aspects of lipid metabolism, which potentially determines the nutritional benefits of CO.

The greatest number of adults living with chronic kidney disease (CKD) are in China (up to 159.8 million), which imposes great pressure and a grim challenge on national health system of China [22]. The changed fatty acid metabolism in patients with CKD induces mitochondrial dysfunction and cellular damage and contributes to the progression of kidney damage [23]. It is widely recognized that dieting is a highly effective approach for preventing and treating metabolic disorders [24]. The Mediterranean diet, in which extra-virgin olive oil (EVOO) is the main source of vegetal fats, represents a nutritional-diet regimen that is useful for the treatment of CKD [25]. CO is regarded as the “Oriental olive oil.” In the literature and in our previous study, it has been observed that CO intake reduced the kidney index in high-fat-fed rats, suggesting that, besides the liver, the impact of CO on the kidneys is also worthy of attention [26,27]. However, there are few research and review articles that focus on the effects of CO on kidney function.

In this study, a rat model of hyperlipidemia was established. Refined and virgin CO were administered to observe their effects on glycolipid metabolism in the kidneys of rats fed with a high-fat diet.

## 2. Materials and Methods

### 2.1. Preparation of Virgin CO

Fresh camellia fruits were collected from the forestry station of the Hunan Academy of Forestry in October 2022. The harvested fruits were stacked at room temperature for 6 to 7 days, then spread out in the sun for 3 to 4 days to allow them crack naturally. The collected fresh seeds were dried at 40 °C until they reached a constant weight. The CO was squeezed using mall-pressed technologies by applying physical pressure at low temperature (<65 °C) and then filtered using a plate and frame filter [3].

### 2.2. Refining of CO

The standard refining process of pressed CO including the following steps [28,29]:

Degumming: The crude oil was heated to 60 °C. Three percent (*v*/*v*) soft water at 80 °C was added to the oil. The mixture was stirred for 30 min and then centrifuged at 3000 rpm, 55 °C for 20 min to separate the oil and gum.

Neutralization: 1.5% (*v*/*v*) NaOH solution (15–25% *w*/*v*) was added to the crude oil, and the mixture was stirred continuously for 2 h and heated slowly to 60 °C to form soaps. The mixture was then left to stand for 6–8 h to allow the generated soaps to precipitate completely, after which the soap was discharged.

Washing: Fresh water was added to the oils. The mixture was stirred for 4 h and heated slowly to 90 °C. The aqueous layer was then removed, and fresh water was added. After this process was repeated 2–3 times, the temperature was raised to 105 °C, and the oil was stirred for 1 h to dehydrate the oils until the water content was reduced to less than 0.1%.

Decolorization: 4% activated clay (*w*/*v*) was added to the washed oil. The mixture was stirred for 30 min at a pressure of 0.1 MPa and a temperature of 90 °C. Then, the oil temperature was cooled to 70 °C and filtered to remove the clay.

Deodorization: Deodorization was performed at 240 °C for 4 h at the pressure of 190 Pa.

Winterization: The CO was slowly stirred and cooled to 4 °C, then maintained at 4 °C for 18 h to allow the growth of crystals. The cooled and crystallized oil sample was then filtered to obtain the refined CO.

### 2.3. Determination of Fatty Acid Profile

The fatty acid composition of the CO was analyzed through methyl esterification. The oil was converted into methyl esters using potassium hydroxide-methanol and extracted using *n*-hexane. The oil phase was then washed with distilled water and dried with anhydrous sodium sulfate. The analysis was conducted using a Shimadzu GC2014 gas chromatograph equipped with a SP2340 capillary column (60 m × 0.25 mm × 0.2 μm) (Supelco, Bellefonte, PA, USA) and a flame ionization detector (FID) [30]. Approximately 1 μL (split 1:100) of *n*-hexane phase was injected. The injection and detection temperatures were set at 250 °C and 260 °C, respectively. After injection, the column temperature was initially held at 50 °C for 2 min and then ramped up at a rate of 10 °C/min to 170 °C, at which it was maintained for 10 min. Subsequently, the temperature was increased by 2 °C/min to 180 °C, with a retention time of 10 min. Finally, the temperature was further increased by 4 °C/min to 220 °C, with a retention time of 22 min. Nitrogen was used as the carrier gas (0.65 mL/min). The retention time of each fatty acid was compared to the corresponding standard sample for identification, and the relative content of each fatty acid was determined using peak area normalization. Each value represents the average of the three independent experiments.

### 2.4. Nutritional Substance Determination

The content of tocopherols was analyzed following the previously described method. A 1.0 g oil sample and 0.1 g of BHT were dissolved together in *n*-hexane to obtain a 25 mL solution. The analysis was performed using a Shimadzu LC20A high-performance liquid chromatograph equipped with a Waters Spherisorb ODS2 column (particle size 5 μm, 4.6–150 mm) [31]. Separation was carried out using a mobile phase consisting of methanol and water (96:4, *v*/*v*) with a flow rate of 2 mL/min. The analytical column was maintained at a temperature of 45 °C. Detection was set at 292 nm.

To determine the total polyphenols, the oil sample was dissolved in *n*-hexane and then purified using a glycol-based column. The Folin-phenol reagent was used to determine the total polyphenols, and the absorption was measured at a wavelength of 750 nm. The polyphenol concentration was calculated based on the standard curve of gallic acid [32].

To determine the qualene and phytosterol levels, a 0.4 g oil sample was added to a conical bottle along with 10 mL of KOH-ethanol (2 mol/L). The solution was saponified at 80 °C for 50 min, and the unsaponified substances were extracted with *n*-hexane, washed to neutral, dried, and brought to a volume of 2 mL. Gas chromatography was used to calculate the contents of squalene and phytosterol using the external standard method [33]. The analysis was conducted using an Agilent HP-5 column and a flame ionization detector (FID). The injection port temperature was set at 220 °C, and the injection volume was 1 μL. The column temperature was initially held at 160 °C for 1 min, then increased at a rate of 15 °C/min to 280 °C where it was maintained for 5 min, after which it was increased by 5 °C/min to 300 °C and held for 7 min.

To determine the concentration of proanthocyanidins, a 1 mL oil sample was dissolved in 9 mL of 50% (*v*/*v*) methanol solution. An amount of 1 mL of the diluted solution was added into a 10 mL conical flask, followed by the addition of 6 mL of a butanol-hydrochloric acid (95:5, *v*/*v*) mixture and 0.2 mL of a 2% (*w*/*v*) ammonium iron sulfate solution. The mixture was then refluxed in a boiling water bath for 40 min and immediately cooled in an ice water bath. Afterward, the flask was left at room temperature for 10 min before the absorbance was measured at a wavelength of 546 nm. The concentration of proanthocyanidins was calculated through the standard curve [34].

### 2.5. Animal Studies

Twenty-four male Sprague-Dawley (SD) rats at the age of 6 weeks (180–200 g) were obtained from the Slac Laboratory Animal Co. Ltd. (Changsha, China). A standard rodent diet (12% fat by calories) and a high-fat diet (HF) containing 10% (*w*/*w*) lard oil were provided by the same supplier. The rats were individually housed in cages with free access to food and water and were kept under controlled temperature (22 °C) and lighting conditions (12 h of light and 12 h of dark).

After 1 week of adjustable feeding with the standard rodent diet, the rats were randomly allocated into three treatment groups of eight replicates each and fed with a high-fat (HF) diet. The high-fat diet contained 56% (*w*/*v*) corn, 20% (*w*/*v*) soybean meal, 8% (*w*/*v*) beer yeast, 3% (*w*/*v*) fish meal, 10% (*w*/*v*) lard, 1.5% (*w*/*v*) cholesterol, 0.5% (*w*/*v*) bile acid, and 1% (*w*/*v*) premixture. The HF diet control group (G) received intragastric administration of saline, while the indicated groups were given pressed or refined CO (Z and R, respectively) by gavage (1.5 g/kg) once a day at 5 pm. Rat weights were measured daily at 5 pm. After the completion of the 8-week treatment, all rats were bled from the eyes and then sacrificed. The livers and kidneys were rapidly removed, weighed, and stored in liquid nitrogen.

All animal studies were approved by the Animal Care Committee of the Hunan University of Science and Technology.

### 2.6. Biochemical Analysis

The plasma glucose, total cholesterol (TC), triglycerides (TG), low-density lipoprotein cholesterol (LDL-C), and high-density lipoprotein cholesterol (HDL-C) were measured using commercial kits according to the manufacturer’s instructions (Jiancheng Bioengineering Institute, Nanjing, China). The liver and kidney TAG and TG were extracted using the method described by Bligh and Dyer [35] and determined using commercial kits (Jiancheng Bioengineering Institute, Nanjing, China). The protein concentrations were measured using a BCA protein assay kit (Solarbio, Beijing, China). The ATP content in the livers and kidneys was measured by a commercial kit (Jiancheng Bioengineering Institute, Nanjing, China).

### 2.7. Quantitative Real-Time PCR

The total RNA was extracted from the kidney tissues using TRIzol reagent (Life Technologies Corporation, Carlsbad, CA, USA). The RNA was reverse-transcribed via standard reagents (PrimeScript^RT^ Master Mix, Takara Bio Inc., Dalian, China) and using random primers. The complementary DNA was amplified in a 7500 Fast Real-time PCR System using TB Green Premix Ex Taq (Takara Bio Inc., Dalian, China). The primers used are listed in Table 1. The mRNA expression levels normalized to ubiquitin were expressed using the comparative delta CT method [36].

### 2.8. Western Blot Analysis

The total proteins from the kidney samples were quantified using western blot (WB) analysis, as described previously [36]. The following antibodies were chosen to analyze the level of total proteins: ACC (3662S, 1:1000 Cell Signaling Technology, Beverly, MA, USA), *p*-ACC (3661S, 1:1000 Cell Signaling Technology), HMGCR (PA5-37367, 1:1000 Invitrogen, Carlsbad, CA, USA), PFK (#8164T, 1:1000 Cell Signaling Technology), CPT1 (ab234111, 1:1000 Abcam, Cambridge, MA, USA), SREBP2 (ab30682, 1:1000 abcam), AMPK (5832T, 1:1000 Cell Signaling Technology), and β-actin (4970S, 1:1000 Cell Signaling Technology), along with the HRP-conjugated secondary antibodies (7074S, 1:2000 Cell Signaling Technology).

### 2.9. Oil Red O Staining

The fresh kidney and liver tissues were rapidly frozen in liquid nitrogen and then transferred to −16 to −18 °C for frozen sections. The thickness of the sections was 4–8 μm. After air-drying at room temperature for 15–20 min, the sections were incubated in 100% isopropanol for 5 min, followed by incubation in a 0.5% (*w*/*v*) Oil Red O solution at 60 °C for 7–8 min. The sections were then washed with 85% isopropanol for 3 min, followed by double-distilled water rinses. They were stained with hematoxylin for 1–1.5 min, followed by double-distilled water rinses. Finally, the sections were mounted with glycerol and gelatin, and the samples were observed using a microscope [37].

### 2.10. Statistical Analysis

A two-way analysis of variance (ANOVA) was used to examine the effects of CO on the rats. The statistical significance was evaluated at the *p* < 0.05 level. Data analysis was carried out using SPSS 20.0 (IBM, Chicago, IL, USA).

## 3. Results

### 3.1. Effects of Refining on the Relative Contents of CO

In this study, the virgin CO was refined according to the commonly used method in the production of soybean oil. The results indicated that the dominant fatty acids in CO were palmitic, stearic, oleic, linoleic, and linolenic acid (Table 2). The fatty acid composition of CO was minimally affected by the refining process. The proportion of palmitic acid and stearic acid decreased to some extent (18.87% and 3.59%), while the proportion of oleic acid slightly increased (2.31%). Significant reductions were observed in the content of the primary trace active substances, including sterols, vitamin E, squalene, polyphenols, and procyanidins, with a decrease of 24.92%, 63.55%, 19.59%, 84.74%, and 100%, respectively. These results indicate that, compared to fatty acids, the refining process has a greater impact on the active components.

### 3.2. Virgin CO Alleviated Weight Gain in HF-Fed Rats

The starting body weight of rats in each group was comparable, and there were no significant differences in food intake among the different groups (Table 3). When compared to the high-fat control group (group G), the group treated with virgin CO (group Z) exhibited a significant decrease in final weights, while the group treated with refined CO (group R) did not. Additionally, the use of virgin CO significantly reduced the liver and kidney weights at the same time, whereas the refined CO only reduced the kidney weights. Consequently, the liver and kidney indexes of group Z were significantly lower than those of group G. These findings suggest that CO can improve the fundamental physiological indicators of rats fed with a high-fat diet, with virgin CO being more effective than refined CO.

### 3.3. Virgin CO Alleviated Dyslipidemia in HF-Fed Rats

**Figure 1:**
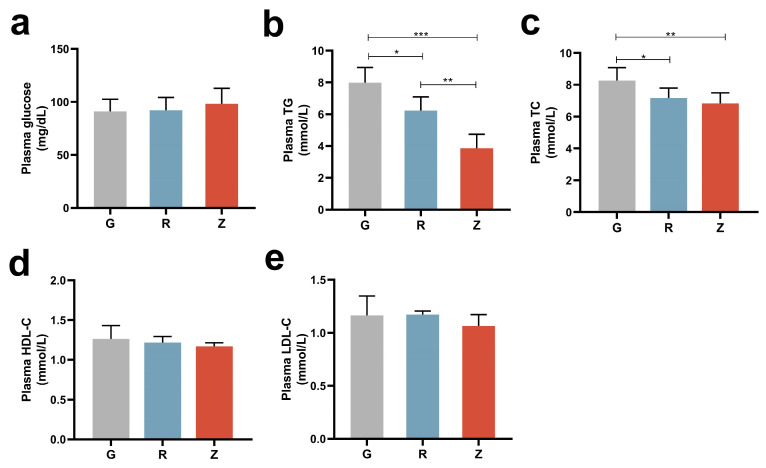
Virgin-CO-mitigated dyslipidemia in HF-fed rats: (**a**) plasma glucose, (**b**) plasma TG, (**c**) plasma TC, (**d**) plasma HDL-C, and (**e**) plasma LDL-C. Mean ± SEM, * *p* < 0.05, ** *p* < 0.01, *** *p* < 0.001.

Figure 1a illustrates the impact of oral CO on the rats’ blood sugar levels. The results indicate that there was no significant difference in blood sugar levels among groups G, R, and Z. Both the R and Z groups showed a significant decrease in plasma TG and TC levels compared to the G group (Figure 1b,c). Moreover, the plasma TG in the Z group decreased significantly as compared to the R group, while the plasma TC showed a slight but not significant decrease. Notably, there were no significant changes observed in the HDL-C and LDL-C levels across all groups (Figure 1d,e). Overall, this study emphasizes the positive effects of CO on the regulation of blood lipids, with virgin CO yielding better results than refined CO.

### 3.4. CO Mitigated Lipid Deposition in Liver

**Figure 2:**
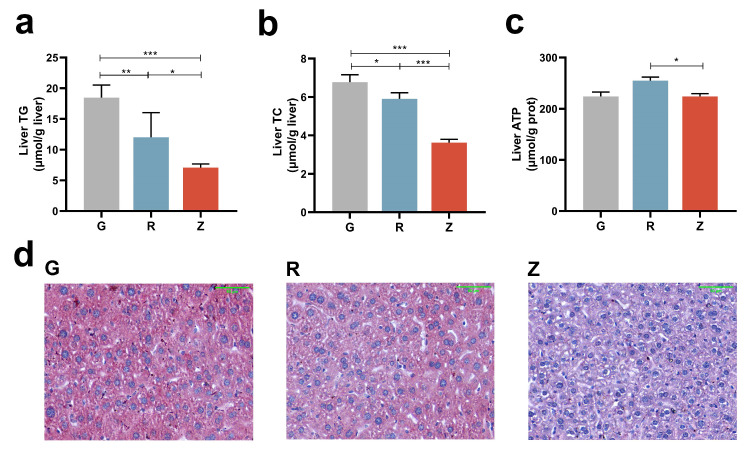
CO-mitigated lipid deposition in liver: (**a**) liver TG, (**b**) liver TC, (**c**) liver ATP content, and (**d**) Oil Red O staining of liver tissues. Magnification: 200×. Scale bar = 50 μm. Mean ± SEM, * *p* < 0.05, ** *p* < 0.01, *** *p* < 0.001.

Figure 2 depicts the levels of TG and TC in the livers of the rats. Remarkably, both the R and Z groups exhibited a significant decrease in TG and TC content when compared to group G (Figure 2a,b). Furthermore, the liver TC and TG contents in group R was notably higher than those of group Z. In terms of the liver ATP contents, group Z did not display any significant difference when compared to group G, whereas group R exhibited a significant increase when compared to group Z (Figure 2c). The Oil Red O staining indicated that the lipid contents in the livers of groups R and Z were considerably lower than those group G, with group Z having lower lipid contents than group R (Figure 2d). These results suggest that CO, particularly virgin CO, plays a pivotal role in regulating lipid accumulation in the liver.

### 3.5. Virgin CO Mitigated Lipid Deposition in Kidney

**Figure 3:**
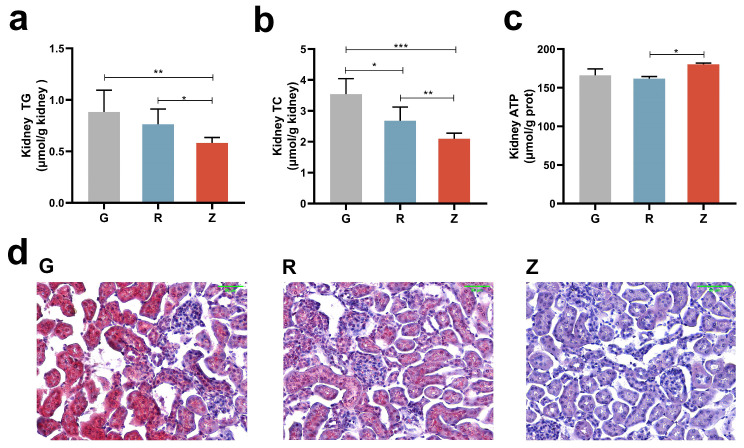
Virgin-CO-mitigated lipid deposition in the rats’ kidneys: (**a**) kidney TG, (**b**) kidney TC, (**c**) kidney ATP content, and (**d**) Oil Red O staining of kidney tissues. Magnification: 200×. Scale bar = 50 μm. Mean ± SEM, * *p* < 0.05, ** *p* < 0.01, *** *p* < 0.001.

Figure 3 depicts the levels of TG and TC in the kidneys of the rats. Remarkably, both the R and Z groups showed a significant decrease in TC levels compared to group G (Figure 3a,b). Additionally, the TC and TG levels in the kidneys of group R were notably higher than those of group Z. In terms of the ATP contents in the kidneys, group R did not exhibit any significant difference compared to group G, whereas group Z showed a significant increase in comparison to group R (Figure 3c). The Oil Red O staining revealed that the lipid content in the kidneys of group R and Z was considerably lower than that of group G, with group Z having a lower lipid content than group R (Figure 3d). These findings suggest that CO, especially virgin CO, plays a crucial role in mitigating lipid deposition in the kidney.

### 3.6. Virgin CO Reduced Lipid Synthesis in Kidney

**Figure 4:**
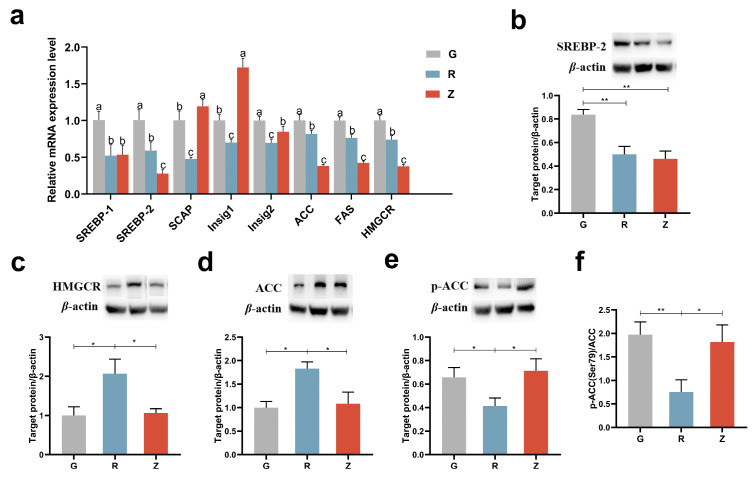
Virgin-CO-reduced lipid synthesis in the rats’ kidneys: (**a**) mRNA levels of genes involved in lipid synthesis, (**b**) renal expression of SREBP-2, (**c**) renal expression of HMGCR, (**d**) renal expression of ACC, (**e**) renal expression of *p*-ACC, and (**f**) phosphorylation level of ACC. Mean ± SEM, * *p* < 0.05, ** *p* < 0.01. The different superscript letters indicate significant differences among the samples at the level of 0.05.

The mRNA levels of the genes involved in the lipid synthesis in the kidney were examined (Figure 4a). It is noteworthy that the expression levels of Sterol-regulatory element binding protein-1 (SREBP-1) and Sterol-regulatory element binding protein-2 (SREBP-2) were significantly reduced following the CO administration. Moreover, the expression of SREBP-2 in group Z was significantly lower as compared to group R. The SREBP cleavage-activating protein (SCAP), insulin-induced gene-1 (Insig1), and insulin-induced gene-2 (Insig2) are key regulatory factors in the maturation of SREBPs [36]. In comparison to group G, the expression levels of SCAP, Insig1, and Insig2 were significantly reduced in group R. Conversely, the expression levels of SCAP and Insig1 in group Z were significantly increased as compared to group G. Furthermore, the expression levels of SCAP, Insig1, and Insig2 were all higher in group Z than in group R. Acetyl-CoA carboxylase (ACC), Fatty acid synthetase (FAS), and HMG-CoA reductase (HMGCR) are the pivotal enzymes involved in fatty acid and cholesterol synthesis [36]. Significantly decreased expression levels of ACC, FAS, and HMGCR were observed after the CO treatment. Compared to group Z, there was a significant increase in the expression of ACC, FAS, and HMGCR in group R.

Western blot (WB) analysis indicated that the protein content of SREBP-2 was significantly reduced in group R as compared to group G (Figure 4b). Consistent with the mRNA levels, the protein contents of SREBP-2 was lower in group Z than in group G. However, group R exhibited higher protein contents of ACC and HMGCR as compared to group G (Figure 4c,d). Similarly, the protein contents of ACC and HMGCR were lower in group Z than in group R. Moreover, the protein content of phosphorylated ACC in group R was lower than in groups G and Z (Figure 4e). When calculated, the phosphorylation level of ACC indicated a much lower level of phosphorylation in group R as compared with groups G and Z (Figure 4f). Overall, the virgin CO administration downregulated the genes responsible for lipid synthesis.

### 3.7. Virgin CO Enhanced Fatty Acid Oxidation in Kidney

**Figure 5:**
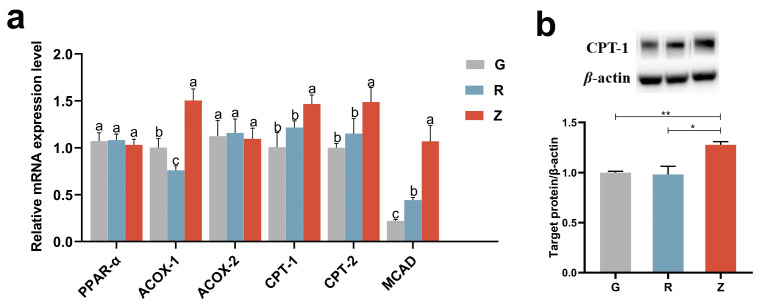
Virgin-CO-tenhanced fatty acid oxidation in the rats’ kidneys: (**a**) the mRNA levels of genes involved in fatty acid oxidation and (**b**) the renal expression of CPT-1. Mean ± SEM, * *p* < 0.05, ** *p* < 0.01. The different superscript letters indicate significant differences among the samples at the level of 0.05.

The effect of refining on the key enzymes involved in fatty acid oxidation is shown in Figure 5. Compared with group G, the expression of acyl-CoA oxidase-1 (ACOX1), the rate-limiting enzyme of peroxisomal fatty acid β-oxidation (FAO), significantly decreased in group R [36]. Conversely, the expressions of ACOX1, medium-chain acyl-CoA dehydrogenase (MCAD), carnitine palmitoyltransferase-1 (CPT-1), and carnitine palmitoyltransferase-1 (CPT-2) significantly increased in group Z. Moreover, the expression levels of ACOX1, CPT-1, and CPT-2 in group R were significantly lower than those in group Z. There were no significant differences in the expression levels of acyl-CoA oxidase-1 (ACOX2) and the peroxisome proliferator activator receptor α isoform (PPARα) among different groups. WB analysis revealed a significant increase in the protein contents of CPT-1 in group Z as compared to groups G and R (Figure 4b).

### 3.8. Virgin CO Enhanced Glucolysis in Kidney

**Figure 6:**
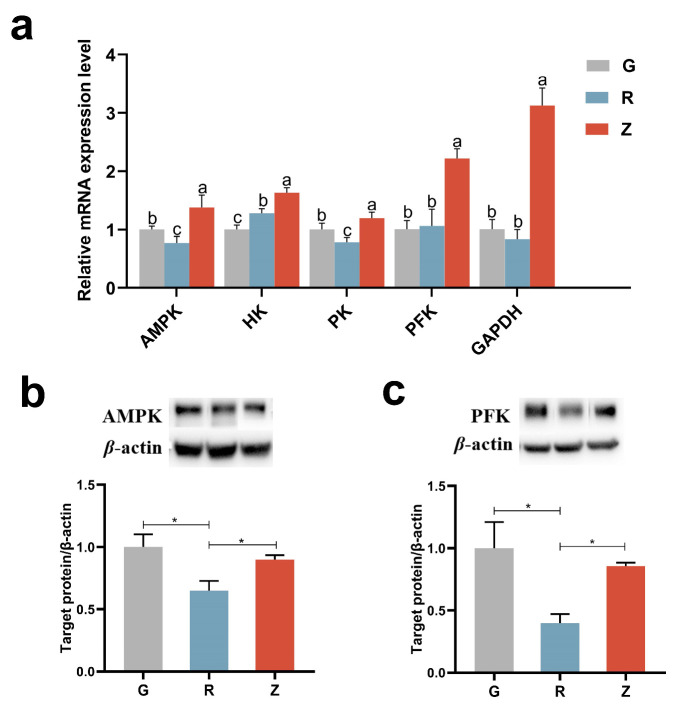
Virgin-CO-enhanced glucolysis in the rats’ kidneys: (**a**) mRNA levels of genes involved in glucolysis, (**b**) renal expression of AMPK, and (**c**) renal expression of PFK. Mean ± SEM, * *p* < 0.05. The different superscript letters indicate significant differences among the samples at the level of 0.05.

As shown in Figure 6, compared with group G, the AMP-activated protein kinase (AMPK) expression level in group R was significantly reduced. The effect of refining on key enzymes in glucolysis is also shown in Figure 6a. Compared with group G, the expression of hexokinase (HK) in group R significantly increased, while the expression of pyruvate kinase (PK) significantly decreased, and the expression of phosphofructokinase (PFK) and glyceraldehyde phosphate dehydrogenase (GAPDH) did not change. The expression levels of HK, PK, PFK, and GAPDH in group Z were significantly higher than those in group G and R.

WB analysis indicated that the protein contents of AMPK and PFK were significantly reduced in group R as compared to group G (Figure 6b,c). Consistent with the mRNA levels, the protein contents of AMPK and PFK were higher in group Z than in group R. Overall, virgin CO upregulated the genes responsible for glucolysis.

## 4. Discussion

### 4.1. Effects of Refining on the Nutritional Ingredients of CO

CO is a natural woody edible oil with a high nutritional value. The nutritional value of CO is primarily attributed to its excellent fatty acid composition and its abundance of trace active ingredients [4,5]. The refinement process plays a crucial role in improving the quality of CO, primarily by eliminating impurities and improving its physicochemical properties [15]. However, the current technological flowsheet used in oil processing plants tends to over-refine the oil, resulting in reduced levels of bioactive compounds in commercial oils [15]. The content of monounsaturated fatty acids in CO is even higher than that found in olive oil [38]. Oleic acid is able to bind to the TLR4–MD-2 complex, inhibit the TLR4-signaling cascades, and ameliorate LPS-induced acute kidney injury [39]. In general, the refining process has a minimal impact on the fatty acid composition of oils like oleic acid (Table 2) [13,14]. One exception is the winterization step, in which saturated fatty acids such as palmitic acid have a higher tendency to crystallize at low temperatures and are therefore filtered out [14].

Polyphenolic compounds in CO are excellent natural antioxidants, exhibiting remarkable influence on anti-aging, anti-hyperlipidemia, anti-obesity and anti-diabetic benefits [40]. However, most of the polyphenolic substances are weak acids, which are easily neutralized by alkali and removed from the oil [14]. Proanthocyanidins are thermosensitive substances that are easily destroyed by high temperatures during the refinement process [41]. They are considered as ligands for peroxisome proliferators-activated receptors (PPARs), which are targets for lipid metabolism disorder and insulin resistance therapy [42]. Polyphenolic compounds seem to be fundamental for the health effects exerted by extra virgin olive oil. Among them, hydroxytyrosol, tyrosol, oleacin, and oleocanthal are of particular importance [43]. The nephroprotective effect of 3′,4′-dihydroxyphenylglycol (DHPG), a polyphenolic compound of extra virgin olive oil, was reported [44]. Benzoic acid, *p*-hydroxybenzoic acid, cinnamic acid, catechin, and naringenin were identified as the major phenolic acid compounds in CO [5]. It is reported that the contents of polyphenolic substances in virgin CO and extra virgin olive oil are quite similar [45]. Therefore, the difference in types of polyphenols may be one of the key factors that determine the nutritional differences between CO and olive oil.

VE is easily oxidized, sensitive to alkali, and can be separated from the oil during the vacuum deodorization process along with the distillate. Additionally, alkali and soapstock can adsorb VE, leading to its loss in the oil [14]. Tocopherols have various biological effects, including antioxidant, anti-inflammatory, anti-cancer, and cardiovascular disease prevention properties [46]. The high-temperature vacuum environment of the deodorization process can result in squalene being carried away with the water vapor [14]. Squalene has various biological effects, such as anti-oxidant, anti-inflammatory, anti-atherosclerotic, and anti-neoplastic benefits, both in vivo and in vitro [47]. Throughout the entire refining process of CO, there is a significant decrease in the content of sterols, especially during the deodorization process [14]. Phytosterols have been proven to have various biological benefits such as anti-hyperlipidemia, anti-obesity and anti-diabetic effects [48].

Overall, it is noteworthy that virgin CO contains various bioactive ingredients, all of which have great potential for combatting the hyperlipidemia associated with a high-fat diet. Consequently, to assess the impact of CO on glycolipid metabolism, rats fed with a high-fat diet were administered either virgin or refined CO.

### 4.2. Effects of CO on Glycolipid Metabolism in the Kidney of Rats Fed with High Fat Diet

Oral CO relieved lipid accumulation in the kidneys and livers of rats fed with high-fat diet and reduced body and kidney weight gain (Figure 2 and Figure 3, Table 3). Comparatively, the effects of virgin CO on reducing body, liver, and kidney weight, as well as mitigating hyperlipidemia and lipid deposition in organs, were more pronounced than those of refined CO. Additionally, the treatment with virgin CO significantly increased kidney ATP content, indicating improvements in energy homeostasis in the kidneys. Therefore, the bioactive ingredients in virgin CO may play a significant role in producing a lipid-lowering effect [1].

In previous reports, CO was found to ameliorate acute kidney injury [39,49]. In this study, we also found that the kidneys are susceptible to the effects of CO intake. This may be attributed to the unique energy source of the kidneys. In the kidneys, mitochondrial fatty acid β-oxidation is a major contributor to ATP production (>60%), which is crucial for cellular activities [50,51]. Specifically, the proximal tubules of the kidneys have high energy demands but relatively weak glycolytic capacities [52,53]. The uptake of free fatty acids (FFA) by the kidneys is positively correlated with their plasma concentration. Unbound FFA can enter the proximal tubule cells of the kidneys via fatty acid translocase and fatty acid binding protein through endocytosis of the apical membranes in a bidirectional manner [54,55].

The presence of hyperlipidemia will increase the transport of fatty acids to the kidneys and upregulate the Cluster of Differentiation 36 (CD36), leading to the enhanced uptake of FFA and the subsequent accumulation of fatty acids in the mitochondria [56]. Fatty acid synthesis is activated in renal cells to generate triglycerides [57]. In diabetic mouse models, the expression of the genes associated with fatty acid synthesis, such as SREBPs, FAS, and ACC, is elevated in the kidneys [58]. Knocking out the gene that mediates CD36 synthesis in corresponding animal models reduces renal lipid accumulation and decreases the likelihood of renal injury and related complications [59]. The long-term elevation of fatty acid levels within the cells can activate NADPH oxidase activity and cause mitochondrial dysfunction, resulting in incomplete beta oxidation and the generation of reactive oxygen species (ROS), thereby inducing oxidative stress in the kidneys [60]. Furthermore, high blood glucose, hyperinsulinemia, and low adiponectin levels notably decrease the activation of the AMPK signaling pathway, thereby reducing fatty acid oxidation [57,61,62,63].

In conclusion, hyperglycemia and hyperlipidemia lead to decreased levels of fatty acid oxidation and increased levels of fatty acid synthesis simultaneously, resulting in renal lipid accumulation [63]. The excessive accumulation of lipids in the kidneys can lead to lipotoxicity, which stimulates multiple signaling pathways that induce oxidative stress, inflammation, fibrosis, endoplasmic reticulum stress, and cell apoptosis, thereby exacerbating renal function impairment [60,64]. Several reports have suggested that diabetic nephropathy may be associated with abnormal renal lipid metabolism, a suggestion that has been confirmed in multiple animal models and human experimental studies [64,65].

In this study, we examined the expression of the prominent genes related to glucose and lipid metabolism as well as their regulatory factors (Figure 4, Figure 5 and Figure 6). Our results revealed the virgin CO group exhibited elevated levels of AMPK in the kidneys of rats as compared to the refined CO group. AMPK plays a crucial role in regulating the cellular energy metabolism in the kidneys [57,61,62,63]. Its elevation leads to the inhibition of SREBP-1 and SREBP-2 activities, which are involved in the regulating of fatty acid and cholesterol synthesis, respectively [57,61,62,63]. Also, oleic acid inhibits SREBP processing as well; thus, the mRNA levels of ACC, FAS, and HMGCR are downregulated by CO treatment [66]. Additionally, AMPK can upregulate the expression of Insig1, which serves as a key regulatory factor for SREBPs maturation [67]. Moreover, Insig1 can accelerate the degradation of HMGCR and decrease its protein content [68]. Therefore, the decreased expression of Insig1 in group R could contribute to the high content of the HMGCR protein despite the relatively low mRNA level (Figure 4).

AMPK activation leads to the phosphorylation and subsequent inactivation of various metabolic enzymes (Figure 4). For example, it inhibits the synthesis of cholesterol and TG by phosphorylating and inactivating ACC and HMGCR [69]. In addition to directly regulating key enzymes in these pathways, AMPK also modulates the cellular metabolism by targeting transcriptional regulatory factors [70]. In this experiment, the expression levels of the key enzymes for fatty acid oxidation, such as ACOX1, CPT-1, and CPT-2, were upregulated. Enhanced β-oxidation of fatty acids can improve lipid disorders. Furthermore, AMPK activation can also accelerate glycolysis, primarily through the regulation of key enzymes in the glycolysis process [71]. The results of this experiment showed that the expression levels of key enzymes in glycolysis, such as PFK, HK, PK, and GAPDH, were upregulated [72].

**Figure 7:**
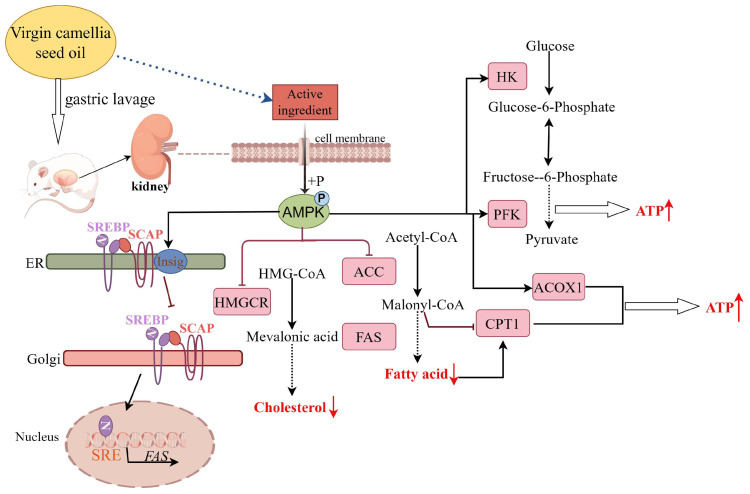
Proposed mechanism by which the virgin CO regulated the glycolipid metabolism through the AMPK-SREBP pathway in the kidneys of rats fed with a high-fat diet (diagram by Figdraw 2.0).

In conclusion, the AMPK activation induced by virgin CO administration can switch on catabolic pathways, such as fatty acid oxidation and glycolysis, to generate ATP and can switch off anabolic pathways, such as fatty acid synthesis. Therefore, virgin COalleviates dyslipidemia and lipid deposition in the kidneys. Chronic administration of small molecule activators elevates the expression and phosphorylation of AMPK in the kidneys and slows down the progression of chronic kidney disease [61,73]. The literature reports have indicated that CO contains direct or indirect activators of liver AMPK [26]. Hydroxytyrosol, a major phenolic substance in virgin olive oil was reported to activate AMPK [74]. It is noted that the polyphenols contained in virgin CO, like benzoic acid and gallic acid, could also activate AMPK, while their contents in refined oil decrease sharply, which partly explains the different regular effects of refined and virgin CO [5,75,76]. Our research demonstrated the presence of activators for kidney AMPK in virgin CO. The proposed mechanism by which the virgin CO regulated the glycolipid metabolism through the AMPK-SREBP pathway in the kidneys of rats fed with a high-fat diet is shown in Figure 7. However, other substances in edible oils can also directly or indirectly activate the AMPK signaling pathway [77,78,79]; thus, the specific substances responsible for AMPK activation in virgin CO have not been identified.

## 5. Conclusions

In conclusion, this study investigated the regulation of renal glucose and lipid metabolism by both virgin and refined CO. It was found that refining has minimal impact on the fatty acid composition of CO, suggesting that the active substances present in virgin CO may be crucial in regulating glucose and lipid metabolism. We propose that the active substances in CO regulated glycolipid metabolism through the AMPK-SREBP pathway in the kidneys of rats fed with high fat diet. To further explore the molecular mechanisms of these active substances in CO, it is necessary to purify and identify them in future research. Additionally, investigating the changes in the active substances in CO during the different processing stages is important for understanding the impact of these techniques on the nutritional value of CO. In consideration of the large number of adults living with CKD in China, further clinical research should be conducted to verify the kidney regulatory function of virgin CO.

## Figures and Tables

**Table 1:** Primers used in this study.

Genes	Forward Primer	Reverse Primer
*ampk*	CAGCCTGACACCTATGG	AATCGGAGCTATCTTCTAG
*srebp1*	GGCACTAAGTGCCCTCAACCT	TGCGCAGGAGATGCTATCTCCA
*srebp2*	CAAGTCTGGCGTTCTGAGGAA	ATGTTCTCCTGGCGAAGCT
*acc*	CCCAGCAGAATAAAGCTACTTTGG	TCCTTTTGTGCAACTAGGAACGT
*hmgcr*	AGCCGAAGCAGCACATGAT	CTTGTGGAATGCCTTGTGATTG
*fas*	CCTGGATAGCATTCCGAACCT	AGCACATCTCGAAGGCTACACA
*ppar-α*	CTGCAGAGCAACCATCCAGAT	GCCGAAGGTCCACCATTTT
*cpt-1*	GGTCTTCTCGGGTCGAAAGC	TCCTCCCACCAGTCACTCAC
*cpt-2*	CAATGAGGAAACCCTGAGGA	GATCCTTCATCGGGAAGTCA
*mcad*	GATCGCAATGGGTGCTTTTGATAGAA	AGCTGATTGGCAATGTCTCCAGCAAA
*acox1*	CTTGGATGGTAGTCCGGAGA	TGGCTTCGAGTGAGGAAGTT
*acox2*	TACCAACGCCTGTTTGAGTG	TTTCCAGCTTTGCATCAGTG
*insig1*	ATGTATCGCGGTGTTTGTTG	TCGATCAAACGTCCACCA
*insig2*	CGCTCTTGGTTGCCATGTA	TGATGAGATTTTTGAGCAATAACTTT
*scap*	TGGGTTGAGGAATGTGTTGGTG	GAAGTAGCCGATGAGGATGATG
*pfk*	CCATGTTGTGGGTGTCTGAG	ACAGGCTGAGTCTGGAGCAT
*hk*	GGAGCAGTGGACCAGGGTA	GAAGTTCAGCTGTTTTTGAATTG
*gapdh*	ACCCTTAAGAGGGATGCTGC	CGGGACGAGGAAACACTCTC
*pk*	AAGAAGGGAGCCACTCTGAA	CTTGTAGTCCAGCCACAGGAT
*ubiquitin*	GCCCAGTGTTACCACCAAGAAG	GCTCTTTTTAGATACTGTGGTGAGGAA

**Table 2:** Effects of refining on the relative contents of CO.

Components	Virgin CO	Refined CO
Palmitic acid (C16:0)	9.22%	7.48%
Stearic acid (C18:0)	1.95%	1.88%
oleic acid (C18:1n9c)	78.80%	80.62%
linoleic acid (C18:2n6c)	8.84%	8.91%
Linolenic acid (C18:3n3)	0.31%	0.30%
Sterols (mg/kg)	2334.88	1753.05
Vitamin E (mg/kg)	104.60	38.13
Squalene (mg/kg)	166.40	133.80
Polyphenols (mg/kg)	346.27	52.83
Proanthocyanidins (mg/kg)	40.00	ND

ND: not detected.

**Table 3:** Fundamental physiological indexes of the rats.

Physiological Indexes	G	R	Z
Food intake (g/d)	24.30 ± 0.31	24.91 ± 0.51	24.97 ± 0.27
Starting weight (g)	251.34 ± 6.88	249.57 ± 7.23	250.12 ± 7.01
Final weight (g)	501.17 ± 9.48 ^a^	497.65 ± 9.59 ^a^	474.96 ± 14.39 ^b^
Liver weight (g)	22.79 ± 0.61 ^a^	21.59 ± 1.31 ^a^	18.83 ± 0.90 ^b^
Kidney weight (g)	2.91 ± 0.15 ^a^	2.64 ± 0.08 ^b^	2.47 ± 0.14 ^c^
Liver index (g liver/100 g BW)	4.55 ± 0.26 ^a^	4.34 ± 0.13 ^a^	3.97 ± 0.12 ^b^
Kidney index (g kidney/100 g BW)	0.58 ± 0.07 ^a^	0.53 ± 0.02 ^b^	0.52 ± 0.05 ^b^

Different superscript letters indicate significant differences among the samples at the level of 0.05.

## Data Availability

The data that support the findings of this study are available from the corresponding author upon reasonable request.

## Abbreviations

HF, high fat; CO, camellia seed oil; TC, total cholesterol; TG, Total triglycerides; HDL-C, High-Density Lipoprotein Cholesterol; LDL-C, Low-Density Lipoprotein Cholesterol; RT-PCR, quantitative real-time PCR; FAS, Fatty acid synthetase; HMGCR, HMG-CoA reductase; SREBP, Sterol-regulatory element binding protein; SCAP, SREBP cleavage-activating protein; Insig, insulin-induced gene; ER, endoplasmicreticulum; HK, hexokinase; PFK, phosphofructokinase; PK, pyruvate kinase; GAPDH, glyceraldehyde phosphate dehydrogenase gene; PPARα, peroxisome proliferator activator receptor α isoform; CPT1, carnitine palmitoyltransferase-1; CPT2, carnitine palmitoyltransferase-2; MCAD, medium-chain acyl-CoA dehydrogenase; ACOX1, acyl-CoA oxidase-1; ACOX2, acyl-CoA oxidase-2; FAO, fatty acid oxidation; ACC, acetyl-CoA carboxylase; AMPK, AMP-activated protein kinase; VE, vitamin E; SD rats, Sprague-Dawley rats; FFA, free fatty acids; CKD, Chronic kidney disease.
